# The Gut Microbiome in Anorexia Nervosa: Friend or Foe?

**DOI:** 10.3389/fpsyt.2020.611677

**Published:** 2021-01-12

**Authors:** Ana Ghenciulescu, Rebecca J. Park, Philip W. J. Burnet

**Affiliations:** ^1^Oxford Medical School, Medical Sciences Division, University of Oxford, Oxford, United Kingdom; ^2^Department of Psychiatry, University of Oxford, Oxford, United Kingdom

**Keywords:** starvation, microbiota, gut-brain axis, metabolites, dysbiosis, treatment

## Abstract

The human gut microbiome is emerging as a key modulator of homeostasis, with far-reaching implications for various multifactorial diseases, including anorexia nervosa (AN). Despite significant morbidity and mortality, the underlying mechanisms of this eating disorder are poorly understood, but the classical view defining AN as a purely psychiatric condition is increasingly being challenged. Accumulating evidence from comparative studies of AN and healthy fecal microbial composition reveals considerable low divergence and altered taxonomic abundance of the AN gut microbiome. When integrated with preclinical data, these findings point to a significant role of the gut microbiome in AN pathophysiology, via effects on host energy metabolism, intestinal permeability, immune function, appetite, and behavior. While complex causal relationships between genetic risk factors, dietary patterns and microbiome, and their relevance for AN onset and perpetuation have not been fully elucidated, preliminary clinical studies support the use of microbiome-based interventions such as fecal microbiota transplants and probiotics as adjuvants to standard AN therapies. Future research should aim to move from observational to mechanistic, as dissecting how specific microbial taxa interact with the host to impact the development of AN could help design novel therapeutic approaches that more effectively address the severe comorbidities and high relapse rate of this serious disorder.

## Introduction

Anorexia nervosa (AN) is a complex and debilitating eating disorder characterized by a distorted body image and extreme dietary restriction, accompanied by severe weight loss and high psychiatric comorbidity (1). Despite having the highest mortality rate of all psychiatric disorders (2), the development of effective, evidence-based treatments has been hindered by a poor understanding of its etiology, which likely involves a complex interplay between genetic and environmental factors (3). Recent evidence from large-scale genome-wide association studies has revealed significant correlations between AN and various metabolic factors, highlighting the importance of reconceptualising AN as a metabo-psychiatric disorder (4, 5). Expanding our horizons beyond a narrow, brain-centric approach to AN might thus provide novel insights into the fundamental mechanisms behind its development, maintenance, and persistence. An emerging avenue of AN research involves the gut microbiome, which is increasingly being acknowledged as a key interface for gene-environment interaction, with important implications for both health and disease (6). The human gastrointestinal tract harbors an abundant and diverse microbial ecosystem, defined as the gut microbiome, forming an evolutionary-driven symbiotic relationship important in maintaining host homeostasis. Over the past decade, advances in metagenomic sequencing technologies and proteomics have enabled the in-depth interrogation of the composition and function of these microbial communities. Compelling evidence supports the involvement of intestinal bacteria in core features of AN, including appetite and weight regulation (7), as well as in its comorbid symptoms, such as altered mood (8) and gastrointestinal symptomatology (9), through a variety of immune, neuroendocrine and metabolic pathways. Moreover, since the composition of these microbial communities is known to be vulnerable to chronic environmental perturbations, such as changes in dietary patterns (10), deviations in the gut microbiome of AN patients from that of healthy individuals are to be expected. Indeed, a growing body of literature points to an association between AN and profound gut microbiome imbalances. This review will evaluate these preliminary clinical findings which, when integrated with evidence from various animal models, shed light on dynamic changes in host-microbiome interactions that may play protective or detrimental roles over the course of AN and may inform future intervention strategies.

## Dysbiosis in AN Patients

Several cross-sectional studies have revealed microbial alterations when comparing stool samples from AN patients and healthy, normal-weight participants (summarized in Table 1). However, results reported so far have been heterogenous, and difficult to interpret. This may be explained, in part, by differences in the methods used to characterize the microbiota (sampling, analysis techniques, statistical tests). Likewise, factors that may have contributed to compositional differences, such as patient dietary habits and the severity/duration of AN, were not consistently reported throughout the studies. Three investigations (14, 17, 19) found a reduction in α-diversity (measuring within-sample microbial diversity) in AN patients compared to controls. Loss of bacterial diversity is considered a hallmark of dysbiosis and is a recurrent finding in other related disease states, such as undernutrition (21), obesity (22), and IBD (23). Alcock et al. (24) hypothesized that a less diverse gut ecosystem may favor the predominance of “pathogenic” bacterial species, which would have more resources available to manipulate host dietary patterns. Interestingly, two of these studies also found that a lower diversity was significantly associated with depressive symptoms (17), as well as anxiety and eating disorder psychopathology (14) in AN. However, this correlation did not translate to subthreshold psychological variation in an independent sample of healthy individuals (25), suggesting that significant shifts in the microbiome-gut-brain axis might be specific to more extreme levels of psychopathology. Since it is difficult to draw conclusions about causality from these associative findings, insights about specific taxonomic differences might be more informative about the relationship between dysbiosis and AN. While not all studies describe the AN microbiota on species level extensively, and some reported findings are divergent, several patterns emerge. Their potential functional relevance is discussed in the sections below.

**Table 1:** Differences in gut microbiome composition in AN patients.

**References**	**Cohort and analysis technique**	**Reported differences in AN compared to healthy controls**			**Correlations with other parameters:**
		**Microbiome structure**	**Microbiome composition**	**Metabolites**	
Armougom et al. (11)	*n* = 9 AN *qPCR*	N/A	↑*M. smithii* ↔ Firmicutes ↔ Bacteroidetes ↔*Lactobacillus*	N/A	**BMI** negatively correlated with *M. smithii*
Million et al. (12)	*n* = 15 AN *qPCR*	↔ Abundance (total)	↑*M. smithii* ↑*E. coli* ↓*Lactobacillus reuteri*	N/A	**BMI:** Negatively correlated with *M. smithii* and *E. coli* Positively correlated with *L. reuteri*
Morita et al. (13)	*n* = 25 (*n* = 11 AN-BP; *n* = 14 AN-R) *23S rRNA* *qPCR* *Liquid chro matography*	↓Abundance (total)	↓ Obligate anaerobes (*Costridium coccoides* group; *Cl. leptum* subgroup; *Bacteroides fragilis* group) ↓*Streptococcus* ↓*Lactobacillus plantarum*	↓acetate ↓propionate	AN-R vs. AN-BP (↔ genus and family level) *C. difficile* only present in AN-BP group ↓*Cl.coccoides* only in AN-R
Kleiman et al. (14)	*n* = 15 AN *16S rRNA analysis*	↓Richness ↓α Diversity	↑ Bacilli ↑ Coriobacteriales ↑*Bifidobacteria* ↓ Clostridia ↓ Faecalibacterium ↓ Anaerostipes	N/A	Levels of **depression, anxiety** and **ED** psychopathology *negatively correlated* with α diversity **Post-weight gain**: ↔α diversity
Mack et al. (15)	*n* = 55 AN (*n* = 44 after weight gain) *16S rRNA* *analysis* *Gas chromatography*	↔ Richness ↔α diversity ↑β diversity ↓α diversity (only in laxative users)	↑ Methanobrevibacter ↑ mucin-degraders *(*Verrucomicrobia*, Bifidobacteria*) ↑ Clostridium clusters I, XI, XVIII ↓ Bacteroidetes ↓ Actinobacteria ↓ butyrate producers (*Roseburia* spp., *Gemminger* spp.)	↔ SCFAs (total) ↓ butyrate (~*Roseburia* spp.)	**Post-weight gain**: ↑β-diversity ↔α diversity Persistent taxonomic differences
Borgo et al. (16)	*n* = 15 AN *16S rRNA* *qPCR* *Gas-liquid chromatography*	↔ Richness ↔α diversity ↔β diversity	↑ Proteobacteria ↑ Enterobacteriaceae ↑*M. smithii* (if detected) ↓ Firmicutes ↓*Ruminococcus* ↓*Clostridium* ↓*Roseburia*	↓ SCFAs (total) ↓propionate ↓ butyrate ↔ isovalerate, isobutyrate	**Anxiety** and **depression** severity *negatively correlated* with butyrate levels **BMI** negatively correlated with *Bacteroides unifiormis*
Mörkl et al. (17)	*n* = 18 AN *16S rRNA*	↓Richness ↓α diversity	↑Coriobacteriaceae	N/A	**Depression** scores *negatively correlated* with α diversity
Speranza et al. (18)	*n* = 10 AN *Gas chromatography*	N/A	N/A	↓propionate ↓ butyrate ↔ acetate	N/A
Hanachi et al. (19)	*n* = 33 AN (undergoing enteral nutrition) *16S rRNA analysis*	↓ Richness ↓α diversity	↑ Enterobacteriaceae ↑*Klebsiella*, ↑*Salmonella* ↓ Firmicutes ↓*Eubacterium* ↓*Roseburia*	N/A	**BMI** *Positively correlated* with Verrucomicrobiaceae, Ruminococcacea *Negatively correlated* with Clostridiales, Eubacteriaceae
Hata et al. (20)	*n* = 10 (AN-R) *16S rRNA analysis*	N/A	↓Bacteroidetes	N/A	FMT to **GF mice** led to impaired weight gain + anxiety and compulsive behavior

*AN-R, restrictive AN; AN-BP, binge-purging AN; SCFA, short-chain fatty acid; GF, germ-free*.

## Gut Microbiota Adaptations to Starvation

A consistent finding across these studies was that AN microbial communities were enriched in the methanogenic archaeon *Methanobrevibacter Smithii* (11, 12, 15, 16) and its abundance was negatively correlated with BMI in two studies (11, 12). This may represent an adaptive response to prolonged caloric restriction, as methanogens can optimize the efficiency of bacterial fermentation (and thus of energy extraction) by metabolizing excess hydrogen from the gut. However, high levels of *M. smithii* have also been shown to slow intestinal transit (26), which may exacerbate symptoms of constipation often present in AN and interfere with weight restoration. Given the chronic caloric restriction and fluctuating food availability characteristic of AN (27), it is not surprising that microorganisms equipped to survive in a challenging, calorie-poor environment are more likely to thrive. In a pioneering study, Smith et al. (28) transferred stools from children with kwashiorkor, an acute form of protein-energetic malnutrition, to germ-free (GF) mice, which developed drastic weight loss and metabolic abnormalities compared to controls receiving transplants from healthy children. This suggested that, gut microbiota profiles during starvation may play a causal role in body weight regulation. While malnutrition secondary to AN develops more chronically, it is plausible that microbial communities selected by a low-energy gut environment might perpetuate AN, possibly by direct effects on weight loss and behavior (7).

Another possibility is that dysbiosis might be a risk factor for AN. A recent review by Mack et al. (29) considered whether microbe compositional changes in AN are the effect of host starvation or are causal, by comparing these profiles with those of other vertebrates under fasting conditions. However, little consistency was found across the literature regarding alterations in taxonomic abundance, which could reflect environmental and dietary differences across geographic regions. Furthermore, changes in relative abundance might not necessarily reveal all aspects of bacterial adaptations to starvation, as shown by a recent proteomic study (30) which demonstrated changes in gut microbiome metabolic activity in a mouse model of AN. Notably, increased levels of a major ATP-producing enzyme, phosphoglycerate kinase, were detected in the feces of mice with activity-based anorexia (ABA) compared to controls and were attributed to the order *Clostridiales*, which may be interpreted as an adaptive increase in energy harvest capacity by these bacteria to meet the demands of the host. To clarify the complex relationships between gut bacteria, starvation, and AN pathophysiology, future human studies should therefore focus on assessing longitudinal changes in the gut ecosystem and potentially include proteomic analyses to interrogate functional alterations of these communities as well.

## Alteration in Bacterial Metabolites

Another important mechanism by which gut bacteria impact host metabolism is the production of short-chain fatty acids (SCFAs) by fermentation of non-digestible carbohydrates. SCFAs, including acetate, butyrate, and propionate, are thought to contribute to up to 10% of human energy requirements (31), and their fecal excretion is known to be reduced by a hypocaloric diet (10). Studies that measured fecal metabolite levels in AN patients reported reduced levels of SCFAs–particularly butyrate–(13, 15, 16, 18), compared to healthy subjects. Additionally, several carbohydrate-fermenting taxa belonging to the Firmicutes phylum (*Roseburia, Clostridium, Eubacterium*) were consistently underrepresented in AN gut microbiota (14–16, 19). In particular, the relative abundance of *Roseburia* spp. was correlated with decreased fecal butyrate levels (15, 16), which in turn were associated with increases in anxiety and depression scores of AN patients in one study (16). Mounting evidence suggests that SCFAs are not only key local regulators of gut homeostasis, but also influence host energy metabolism and appetite by modulating the intestinal production of feeding hormones (7), which may be of direct relevance to AN. However, it is still unclear whether these metabolites can also access neural circuits and directly affect psychopathology, especially since acetate is the only SCFA thought to reach the peripheral circulation (32). Further studies integrating sequencing data with measurements of both fecal and plasma SCFAs will hopefully lead to a more comprehensive understanding of the role that altered microbial metabolite production may play in core and comorbid features of AN.

## Intestinal Permeability and Immune Mechanisms

Decreased availability of colonic butyrate may also lead to an inflammation-mediated disruption of the intestinal barrier (33). Several studies indicate that decreased carbohydrate-fermenters in AN is also accompanied by a shift toward mucin-degrading microorganisms (e.g. *Verrucomicrobia, Bifidobacteria*) (14, 15). The expansion of these slow-growing species is likely favored by the delayed colonic transit commonly occurring in AN (34) and, especially in a nutrient-deficient environment, they survive by digesting the protective intestinal mucus layer. Gut microbiota alterations observed in AN may thus contribute to increased intestinal permeability, facilitating the translocation of bacterial products into the systemic circulation. This can lead to aberrant immune responses, such as the development of a chronic state of low-grade inflammation, which is increasingly acknowledged to contribute to psychiatric pathology (35). Indeed, increased levels of pro-inflammatory cytokines have been found in acute AN (36), but it unclear whether this is a consequence of dysbiosis, or whether other factors, such as starvation-induced stress, upregulate inflammatory pathways and exacerbate intestinal dysfunction (37).

A “leaky gut” is also thought to underlie the elevated risk for autoimmune diseases found in AN (38). Interestingly, increased abundance of autoantibodies against appetite modulating neuropeptides, such as α-melanocyte-stimulating hormone (α-MSH), have been identified in AN (39, 40). Proteomic studies subsequently revealed that ClpB, a heat-shock protein produced by the commensal *Escherichia coli*, has sequence homology with α-MSH (41) and is also elevated in eating disorders (42). This led to the hypothesis of a microbial origin of α-MSH auto-antibodies. These are thought to arise via cross-reactivity with ClpB traversing the intestinal barrier and might play a causative role in AN by interfering with central satiety pathways (43). Indeed, increases in the family Enterobacteriaceae (or *E. coli* specifically) have been detected in stool samples from AN patients (12, 16, 19). However, while increased colonic permeability has been demonstrated in mice with experimentally-induced AN (44), confirmation in human subjects is lacking (45). It therefore remains unknown to what extent gut bacteria-driven molecular mimicry and barrier disruption contribute to the dysregulation of appetite in AN, but it is plausible that this pathway could precipitate characteristic behavioral manifestations, at least in a subset of AN patients (46).

## Mechanistic Insights into Psychobehavioural Symptoms of AN

It is difficult to disentangle cause from effect based on findings from the observational studies described so far, and it remains unclear whether and how the dysbiosis observed in AN patients contributes to pathophysiology. In a recent study, Hata et al. (20) were the first to address this by reconstituting gnotobiotic mice with microbiota derived from AN patients (gAN). Compared to GF mice who received transplants from healthy controls (gHC), gAN mice exhibited poorer weight gain and a lower food efficiency ratio, which was correlated with the abundance of several bacterial genera previously found to be altered in AN patients (*Clostridium; Bifidobacterium*) (13–16). In addition, AN-derived microbiota appeared to induce anxious and compulsive-like behavior in the recipient mice, which were inversely correlated with both the fecal abundance of *Bacteroides* and brainstem 5-HT levels. Both parameters were also lower overall in gAN mice compared to age-matched gHC. Further, the compulsive phenotype was reversed upon probiotic treatment with *B. vulgatus*, a predominant species of the *Bacteroides fragilis* group [reported to be less abundant in AN by Morita et al. (13)]. Although the sample size was small, these initial findings provide more convincing evidence for a causal role of AN-specific microbiota alterations in key traits of the disorder.

AN patients typically display OCD-like behaviors such as obsessionality, compulsivity and anxiety (1) mainly surrounding food and body image. These behaviors are thought to be driven by similar mechanisms in the two disorders, which also share a significant genetic overlap (47), and are alleviated by serotonin-modulating medication (such as SSRIs) in the case of OCD (48). However, while a serotonergic dysfunction is likely also involved in AN pathogenesis (49), SSRIs have limited effectiveness in the treatment of this eating disorder (50), especially in children and adolescents (51). This could be explained by low serotonin metabolism in the acute phase of AN, probably related to a shortage of its amino acid precursor tryptophan as a result of dietary restriction (52). An influential study by Yano et al. (53) demonstrated that intestinal bacteria play a key role in the metabolism of tryptophan by enterochromaffin cells, which accounts for ~90% of the body's serotonin synthesis. Colonization of GF mice with spore-forming *Clostridium* microbes from healthy humans completely restored serum serotonin levels, providing proof-of-principle that serotonin availability can be manipulated upstream, at the level of gut microbiota. However, *Bacteroides* species, including those form the *B. fragilis* group, had no effect on host peripheral serotonin (53). Therefore, concerning the AN transplantation study by Hata et al. it is unlikely that *B. vulgaris* exerted its protective effects against the compulsive phenotype by directly affecting serotonin synthesis. Considering it was administered to gAN mice throughout the weaning period, the probiotic strain probably had collateral effects on the dynamics of the AN-derived microbial ecosystem, shifting its composition toward a more “beneficial” profile. Further research is thus needed to clarify which bacterial species altered in AN contribute to modifications in neurotransmitter availability, whether these effects are indeed related to relevant behavioral manifestations and how to optimally exploit the microbiota to address features of AN refractory to pharmacological treatment.

## Implications for Treatment

These converging preclinical and clinical findings have encouraged a number of interventional studies investigating the potential of microbiome-based therapies for AN patients. Nutritional rehabilitation is the standard treatment for AN, but current refeeding regimens are often accompanied by severe GI distress (such as gastric-duodenal ulcers) and high rates of weight relapse (54). In addition, studies that followed the change in gut microbiome composition of AN patients over the course of renutrition reported that weight gain did not restore bacterial diversity to levels comparable to normal weight controls (14, 15), suggesting that dysbiosis might persist beyond weight recovery and potentially contribute to relapse. Fecal microbiota transplantation (FMT) from healthy donors is currently being explored as a potential strategy for re-establishing a diverse microbiota and improving the outcomes of nutritional rehabilitation. Two recently published case studies (55, 56) of FMT in AN patients showed promising results, with improvements in gut barrier function, microbiota composition and weight gain, and further FMT studies in larger cohorts are underway. However, the underlying assumption of FMT and its therapeutic potential remains a topic of debate, mainly as there is still no consensus over what constitutes a “healthy” microbiota profile. As the optimal composition is probably different for each individual (57), extensive population-based evidence describing microbiota variations in health and disease will be necessary before establishing appropriate screening parameters of donor-recipient compatibility.

Probiotics are another, less invasive approach for manipulating the gut microbiome, and preliminary clinical evidence suggests that oral administration of particular bacterial strains could have beneficial effects on mood (58) and gastrointestinal function (59). Such results have generated interest in the potential use of probiotic supplementation as a co-adjuvant to standard AN therapy, as they could help tackle comorbidities that often complicate recovery.

## Conclusions

**Figure 1:**
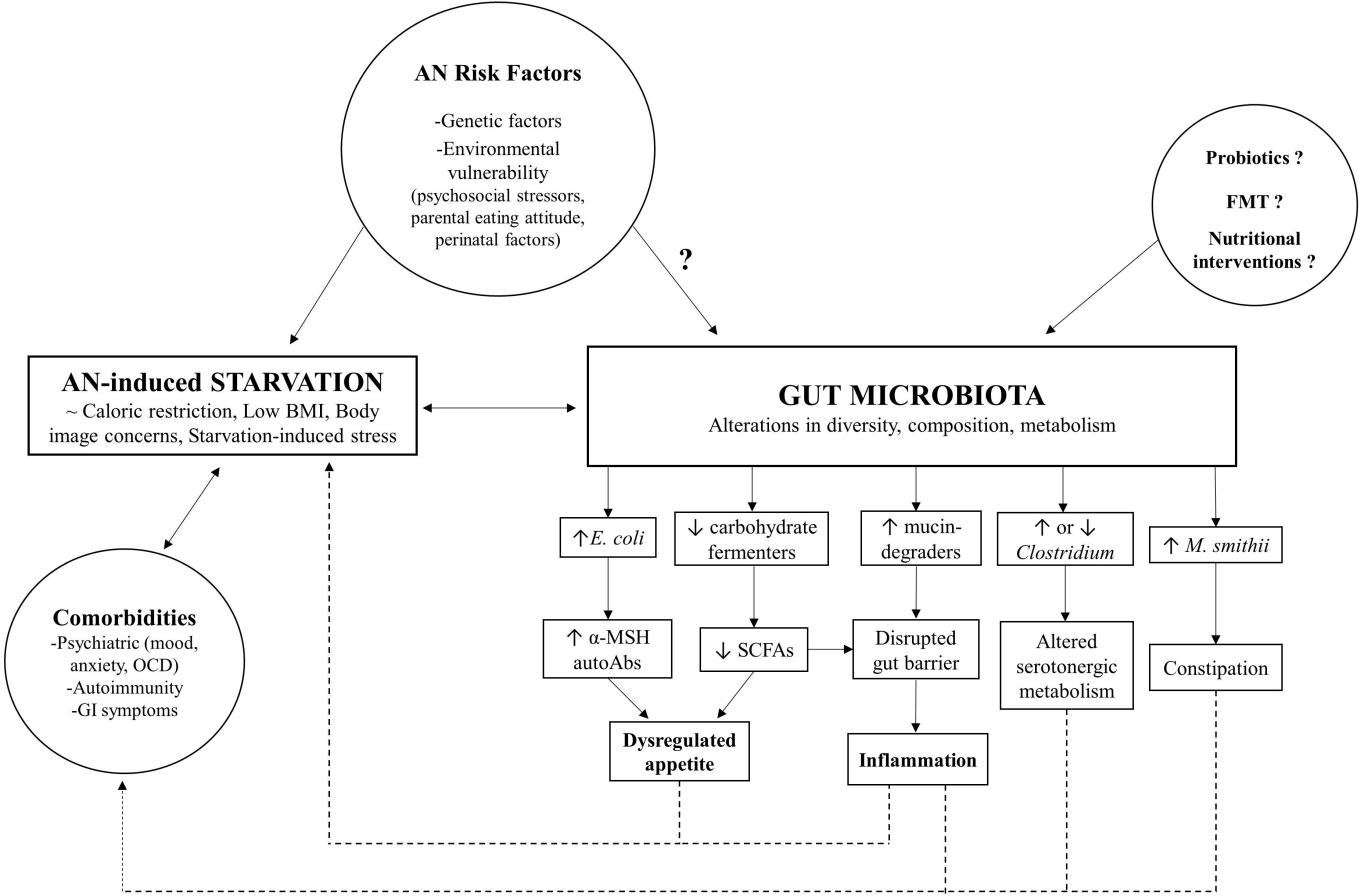
Model for gut microbiome involvement in AN pathophysiology.

Although gut microbiome research in AN is still in its infancy, the evidence supports a key role of intestinal dysbiosis, marked by perturbations in diversity and taxonomic composition, in both somatic and psychological manifestations of this complex, multifactorial disorder (as summarized in Figure 1). However, it remains unclear whether these microbial changes observed in AN patients are a non-specific consequence of chronic caloric restriction, or a causative factor. Microbial adaptations to new, challenging contexts, such as the starvation state characteristic of AN, may occur at the expense of host fitness and act as a reinforcing mechanism for the underlying metabolic dysregulation and comorbidities that impact the course of the disease and complicate treatment. Moreover, findings highlighting the involvement of certain commensal species in the modulation of host appetite, either by direct effects on neuroendocrine secretion or via autoimmune mechanisms, raise the possibility of the gut microbiome directly contributing to the core behavioral phenotype of AN. High quality longitudinal data from AN cohorts, coupled with translational studies transplanting microbiota from anorectic patients to gnotobiotic animals, will be essential steps that will clarify potential causal mechanisms. Preliminary clinical studies serve as proof-of-concept that targeting the microbiome can alleviate AN symptoms and could thus be used as a safe complementary tool to standard nutritional interventions. However, in order to integrate the gut microbiome into a more comprehensive model of AN, a better understanding of how these biological mechanisms interact with psychosocial stressors in at risk individuals is needed. Further exploring the dynamics between gut microbiome, environment and genetics in the context of AN will hopefully guide the development of novel evidence-based interventions that either supplement current therapeutic strategies by enhancing beneficial interactions or interrupt microbe-driven positive feedback mechanisms that may underlie the poor treatment outcomes and chronicity of this debilitating eating disorder.

## Author Contributions

AG and PB made an equal contribution to the conceptualization and drafting of the manuscript and equal contributions to scientific content proof-reading. AG drafted the final version of the document and constructed the accompanying figure and table. RP provided a significant contribution to clinical content and proof-reading. All authors contributed to the article and approved the submitted version.

## Conflict of Interest

The authors declare that the research was conducted in the absence of any commercial or financial relationships that could be construed as a potential conflict of interest.
